# Geographical distribution of *Aedes aegypti* and *Aedes albopictus* (Diptera: Culicidae) and genetic diversity of invading population of *Ae. albopictus *in the Republic of the Congo

**DOI:** 10.12688/wellcomeopenres.14659.3

**Published:** 2018-12-28

**Authors:** Basile Kamgang, Theodel A. Wilson-Bahun, Helen Irving, Michael O. Kusimo, Arsene Lenga, Charles S. Wondji

**Affiliations:** 1 Medical Entomology, Centre for Research in Infectious Diseases, Yaounde, P.O. Box 13591, Cameroon; 2 Faculty of Science and Technology, Marien Ngouabi University, Brazzaville, Congo; 3 Liverpool School of Tropical Medicine, Liverpool, UK

**Keywords:** Aedes albopictus, Aedes aegypti, ecological distribution, arbovirus vectors, genetic diversity, Republic of Congo

## Abstract

**Background: **The arbovirus vector, *Aedes albopictus,* originating from Asia, has recently invaded African countries, including the Republic of the Congo, where it was associated with a chikungunya outbreak. Up until now, little was known about its distribution in relation to the native *Aedes aegypti* and how the invasion will modify the epidemiology of arboviral diseases. Here, we assessed the current distribution of *Ae. albopictus* and *Ae. aegypti* in the Republic of the Congo and explored the genetic diversity of the invading species, *Ae. albopictus*.

**Methods: **Immature stages of *Aedes* were collected in nine locations in the Republic of the Congo in 2017 following a north-south transect and reared to adult stage. Adults were morphologically identified, counted and grouped according to species and location. Genetic diversity of *Ae. albopictus* was assessed by analyzing the cytochrome oxidase I ( *COI*) gene.

**Results:** *Ae.* *albopictus* and *Ae. aegypti* were found together across the country in all the locations investigated. The invasive species is predominant over the native species in all locations except Brazzaville, suggesting that *Ae. albopictus* is displacing *Ae. aegypti* across Congo. When comparing the species distributions across the two largest cities, Brazzaville and Pointe Noire, *Ae. albopictus* was more prevalent than *Ae. aegypti* in the suburbs whereas the opposite situation was reported in the city centre. Mitochondrial DNA analysis revealed very low genetic diversity of *Ae. albopictus* with only three haplotypes recorded across the country supporting the recent introduction of this species in the Republic of the Congo. Phylogenetic tree analysis revealed that *Ae. albopictus* from Congo originated from other tropical Asian countries such as China, likely as a result of increasing trade links.

**Conclusion: **These findings are important for the implementation of vector control strategies and can serve as a foundation for further research on these vectors in the country.

## Introduction

Arthropod-borne viral diseases such as dengue, zika and chikungunya have emerged or re-emerged in several countries of the world during the past decades ^1– 4^.

These viruses are transmitted to vertebrates, including humans, by the bites of infected mosquitoes that share the same ecological niche as the host organism. Indeed, two distinct ecological cycles, enzootic and urban epidemic cycles, have been well documented ^5, 6^. The enzootic cycle occurs in the sylvan environment, involving non-human primates and wild mosquitoes, while urban epidemic cycle occurs in urban environments, implicating human beings and urban mosquitoes such as *Aedes aegypti* Linneaus 1762 and *Ae. albopictus* (Skuse) 1894. Other potential modes of Zika virus transmission to humans have been evoked notably via sexual intercourse or via blood donor ^5^. Both epidemic vectors, *Ae. aegypti* and *Ae. albopictus,* are found in sub-Saharan Africa, where *Ae. aegypti* is native. Two subspecies of *Ae aegypti, Ae. aegypti formosus* and *Ae. aegypti aegypti*, were formally identified by Mattingly in 1957 ^7^. *Ae. aegypti formosus,* is a dark colored mosquito confined to African forests while *Ae. aegypti aegypti* is light-colored with white abdominal scales and is found in human-dominated habitats primarily outside Africa. Generally, *Ae. aegypti* collected in central Africa match *Ae. aegypti formosus* ^8^.

While *Ae. albopictus* is a native of South East Asia, it has now invaded all the five continents during the past 30–40 years ^9, 10^. This rapid global spread has been caused mainly by sales and distribution of used tires across the world ^11^ coupled with the ecological plasticity of the species, enabling its adaptation to various environments ^9^. *Ae. albopictus* was reported for the first time in Central Africa in early 2000 ^12^ and is currently present in almost all central African countries ^13^, where it tends to supplant the indigenous species *Ae. aegypti* in human-domesticated environment ^14, 15^. The predominance of *Ae. albopictus* over *Ae. aegypti* in sympatric areas has been shown to result from the higher mating competitiveness of *Ae. albopictus* over *Ae. aegypti* ^16, 17^. Previous studies in Central Africa showed that both *Ae. aegypti* and *Ae. albopictus* can be found together in the same location and often share the same larval habitats ^14, 15^. In this region, the immature stages of both species develop in stagnant water found mainly in peri-domestic containers such as used tires and discarded tanks. However, in the sympatric area, *Ae. albopictus* prefers containers surrounded by vegetation whereas *Ae. aegypti* prefers containers located in neighbourhoods with high building densities ^15, 18^.

Dengue, Zika and chikungunya were for a long-time considered to be rare in Central Africa, because only sporadic epidemics were reported in the rural environment, with isolation of the viruses in wild mosquitoes and humans ^19, 20^. In the past decades, several outbreaks have been reported in this part of the world, notably a concurrent dengue/chikungunya outbreak in Gabon in 2007, with more than 20, 000 cases of chikungunya ^2^, and a large chikungunya outbreak in 2011 in the Republic of the Congo with more than 11, 000 cases ^21^. This suggests an epidemiological modification of arboviral diseases in the region. During these outbreaks, *Ae. albopictus* was established as the major vector particularly in Gabon ^22, 23^, where Zika was detected in this species ^24^. In Congo, both *Ae. aegypti* and *Ae. albopictus* were found to be positive for chikungunya virus ^25^, implicating both species in virus transmission. This investigation was the first to confirm the presence of *Ae. albopictus* in the Republic of the Congo. Since then, no study has been undertaken to compare the geographical distribution and prevalence of *Ae. aegypti* and *Ae. albopictus* in the Republic of the Congo as well as the genetic diversity of the invading species. Indeed, previous studies in Central Africa based on polymorphisms to the cytochrome oxidase subunit 1 ( *COI*) gene indicated that *Ae. albopictus* populations in Cameroon are related to tropical rather than temperate or subtropical out-groups ^26^. However, the Central African Republic population segregated into two lineages: the first encompassed specimens from tropical areas including all the haplotypes from Cameroon and the second lineage encompassed temperate and subtropical areas ^15^, suggesting multiple sources of *Ae. albopictus*.

To improve entomological surveillance and the control of these arbovirus vectors in the Republic of the Congo, we present here the current nation-wide geographical distribution and prevalence of *Ae. aegypti* and *Ae. albopictus* in this country, and establish the genetic diversity of the invading population of *Ae. albopictus* using the *COI* gene.

## Methods

### Sampling sites

**Figure 1.**
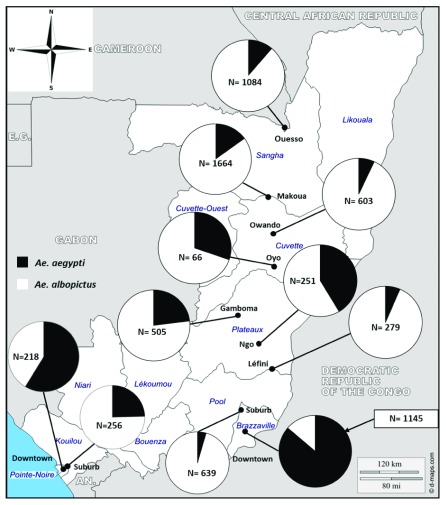
Geographic distribution of *Ae. aegypti* and *Ae. albopictus* across the Republic of the Congo.

Mosquitoes were collected in May and November 2017 (Pointe Noire only) corresponding to the rainy season in nine locations in the Republic of the Congo across the north-south transect ( Table 1 and Figure 1). The Republic of the Congo is located in Central Africa, straddling the equator. Two main types of vegetation are found. The forest in the north, covering 60% of the national territory, and the savannah, which occupies the remaining parts of the country. There are three types of climate. The equatorial climate is found in the north of the country, characterized by high humidity and rainfall greater than 1,700 mm per year, with an average temperature between 24°C and 26°C. The humid tropical climate in the southwest, where annual average precipitation varies from 1,200 mm to 1,700 mm, with an average monthly temperature between 21°C and 27°C. The subequatorial climate, experienced at the plateau and basin regions, has an average annual rainfall of about 1,600 mm. Because the spread of *Aedes* mosquitoes mainly relies on human activities, sampling was focused on human-domesticated environments spread along the main communication networks, and trade routes throughout the country.

**Table 1.** Sampling sites in the Republic of the Congo.

Location	Geographical coordinates	Altitude, m	Climate
Brazzaville	S 4°19'38'' E 15°09'12''	278	Subequatorial climate
Lefini	S 2°54'58" E 15°37'56"	314	Subequatorial climate
Ngo	S 2°29'14" E 15°45'00"	636	Subequatorial climate
Gamboma	S 1°52'27" E 15°52'25"	378	Subequatorial climate
Oyo	S 1°09'14" E 15°58'21"	297	Subequatorial climate
Owando	S 0°29'42" E 15°54'41"	275	Subequatorial climate
Makoua	S 0°00'23" E 19°37'33"	350	Equatorial climate
Ouesso	N 1°36'35" E 16°02'58"	339	Equatorial climate
Pointe Noire	N 4°48'19" E 11°53'23"	14	Tropical climate

### Mosquito collection, rearing and identification

In Brazzaville and Pointe Noire, the two most populated cities of the Republic of the Congo, the difference between downtown and suburban was examined during the investigation. In the other locations, however, samples were collected randomly throughout each city and pooled together. In each selected location, all containers with water were inspected and positive containers (with at least one *Aedes* larva or pupa) were recorded. Immature stages of *Aedes* were collected, transported to the insectaries, pooled according to the location and reared to adult stage for morphological identification. G0 adults were stored at -20°C for molecular and genetic analyses. The comparisons between the prevalence of *Ae. aegypti* and *Ae. albopictus* in each location, across the country were performed using multiple chi-square test.

### Mitochondrial DNA analysis for *Ae. albopictus*

Genomic DNA was extracted from 20 whole *Ae. albopictus* per location (nine locations) using the Livak protocol as previously described ^27^. DNA extracts from each location were used as templates to amplify 700-bp fragment of COI gene. The sequences of primers used are: albCOIF 5’-TTTCAACAAATCATAAAGATATTGG-3’ and albCOIR 5’- TAAACTTCTGGA TGACCAAAAAATCA-3’ ^28^. Polymerase chain reaction (PCR) amplification was performed using a Gene Touch thermal cycler (Bulldog Bio, Portsmouth, USA), as described previously ^28^. PCR products were detected by agarose gel electrophoresis in Tris-Acid-EDTA buffer (TAE). The gel was prepared with Midori green, staining dye, and visualized with the aid of UV light. PCR products from each location with very good amplification were purified using the Exo-SAP protocol and sent to the Centre for Genomic Research (Liverpool, UK) for sequencing.

### Sequence data analysis

Sequences were manually corrected using BioEdit software version 7.2.1 ( http://en.bio-soft.net/format/BioEdit.html) and aligned using ClustalW, which is present in BioEdit ^29^. Sequences were numbered based on the reference sequences downloaded in GenBank KU738429.1. The number of haplotypes (h), the number of polymorphism sites (S), haplotype diversity (Hd) and nucleotide diversity (π) were computed with DnaSP 5.10.01 ( http://en.bio-soft.net/dna/dnasp.html) ^30^. The statistical tests of Tajima ^31^, and Fu and Li ^32^ were also estimated with DnaSP in order to establish non-neutral evolution and deviation from mutation-drift equilibrium. The different haplotypes detected were compared to previous sequences published in GenBank ( Supplementary Table 1) that originated from China, Papua New Guinea, USA, Singapore, Taiwan, Malaysia, Hawai, Christmas Islands, Japan, Solomon Islands, Timor Leste and Torres Strait Islands ^28, 33, 34^. The same *COI* region was sequenced at these various regions and the maximum likelihood phylogenetic tree was constructed using MEGA 7.0 ^35^. Genealogical relationships between haplotype in this current study was assessed using TCS version 1.21 ^36^ and tcsBU ( http://cibio.up.pt/software/tcsBU/) ^37^ software.

## Results

### Containers inspected and prevalence of *Ae. aegypti* and *Ae. albopictus*

A total of 640 containers with water were investigated across the Republic of the Congo ( Table 2). Among them, 42.9% were positive for immature stages of *Aedes*. Containers were classified into three main groups: domestic (flower pot and water storage tanks), peridomestic (used tires, discarded tanks and car wrecks) and natural (axil of plants). Used tires were the most prevalent habitat and most productive containers in all the locations, ranging from 18.1% in Pointe Noire to 100% in Lefini ( Table 2). The presence of *Aedes* in other containers was very limited.

**Table 2.** Containers prospected per location.

Location	Axil of plants	Used tires	Car wrecks	Discarded tanks	Water storages	Flower pots	All
	N (%)	N (%)	N (%)	N (%)	N (%)	N (%)	N (%)
Brazzaville downtown	1 (0.0)	59 (49.2)	3 (100)	10 (80.0)	0 (NC)	0 (NC)	73 (54.8)
Brazzaville suburb	0 (NC)	69 (63.8)	0 (NC)	3 (0.0)	0 (NC)	0 (NC)	72 (61.1)
Pointe Noire downtown	0 (NC)	61 (18.1)	0 (NC)	3 (33.3)	0 (NC)	0 (NC)	64 (18.8)
Pointe Noire suburb	0 (NC)	56 (25.0)	0 (NC)	13 (46.2)	4 (50.0)	0 (NC)	73 (30.1)
Lefini	0 (NC)	3 (100)	0 (NC)	0 (NC)	0 (NC)	0 (NC)	3 (100)
Ngo	0 (NC)	47 (38.3)	0 (NC)	0 (NC)	0 (NC)	0 (NC)	47 (38.3)
Gamboma	0 (NC)	58 (37.9)	0 (NC)	2 (50)	1 (100)	0 (NC)	61 (39.3)
Oyo	0 (NC)	43 (48.8)	0 (NC)	0 (NC)	0 (NC)	0 (NC)	43 (48.8)
Owando	0 (NC)	4 (25)	0 (NC)	8 (62.5)	0 (NC)	10 (10.0)	22 (31.8)
Makoua	0 (NC)	59 (54.2)	0 (NC)	10 (30)	0 (NC)	0 (NC)	69 (50.7)
Ouesso	0 (NC)	97 (50.5)	0 (NC)	0 (NC)	0 (NC)	0 (NC)	97 (50.5)
**All**	**1 (0.0)**	**556 (43.9)**	**3 (100)**	**65 (36.9)**	**5 (60.0)**	**10 (10.0)**	**640 (42.9)**

N, number of containers found with water; (%), percentage of positive containers; NC, not computed.

In total, 6,684 specimens of immature stages of *Aedes* were identified, comprising 72.24% of *Ae. albopictus*, 27.70% of *Ae. aegypti* and 0.06% (four specimens collected in Brazzaville suburb) of *Aedes simpsoni*. *Ae. aegypti* and *Ae. albopictus* were found together in all the locations investigated ( Figure 1 and Table 3). However, *Ae. albopictus* was predominant in all the locations except in Brazzaville. When samples from the two major cities, Brazzaville and Pointe Noire, were divided according to the environment (downtown versus suburb), *Ae. albopictus* was found more prevalent in the suburbs (95.62% and 75.39% in Brazzaville and Pointe Noire, respectively) than *Ae. aegypti*, whereas the reverse was true for the downtown areas ( Table 3).

**Table 3.** Prevalence of *Aedes aegypti* and *Aedes albopictus* according to the location.

Location	*Ae. aegypti*	*Ae. albopictus*	P-value
Brazzaville downtown	962 (86.28%)	153 (13.72%)	<0.001
Brazzaville suburb	28 (4.38%)	611 (95.62%)	<0.001
Pointe Noire downtown	128 (58.72%)	90 (41.28%)	<0.001
Pointe Noire suburb	63 (24.61%)	193 (75.39%)	<0.001
Lefini	18 (6.45%)	261 (93.55%)	<0.001
Ngo	104 (41.43%)	147 (58.57%)	<0.001
Gamboma	116 (22.97%)	389 (77.03%)	<0.001
Oyo	20 (30.30%)	46 (69.70%)	<0.001
Owando	42 (6.97%)	561 (93.03%)	<0.001
Makoua	249 (14.96%)	1415 (85.04%)	<0.001
Ouesso	122 (11.25%)	962 (88.75%)	<0.001
**All**	**1852 (27.72%)**	**4828 (72.28%)**	**<0.001**

### Mitochondrial DNA analysis of *Ae. albopictus*

**Figure 2.**
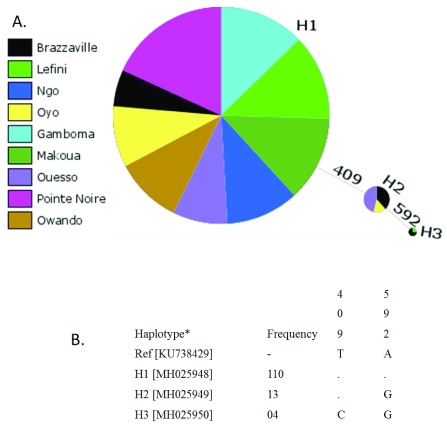
Genetic diversity of the *COI* gene across Congolese populations of *Ae. albopictus*. ( **A**) Haplotype network showing the genealogic relationships between three haplotypes detected across Congo. The pie chart represents the proportion of each haplotype per site. ( **B**) *COI* haplotypes found across the Republic of the Congo. Only polymorphic positions are shown and are numbered with reference (Ref) to the published *Ae. albopictus* sequences for *COI* (JF309317; China). Dots represent identity with respect to the reference. The numbers above nucleotides indicate the position where mutations were found. *GenBank accession number shown in brackets.

**Figure 3.**
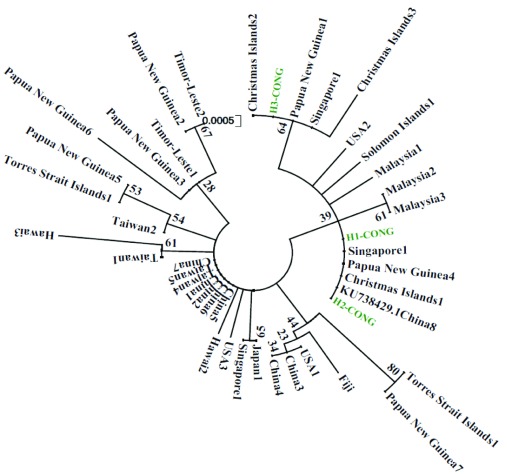
Molecular phylogenetic analysis using the Maximum Likelihood method. The evolutionary history was inferred using the Maximum Likelihood method based on the Tamura 3-parameter model. The tree is drawn to scale, with branch lengths measured in the number of substitutions per site. There was a total of 445 positions in the final dataset.

In total, 127 specimens of *Ae. albopictus* from nine locations across the Republic of the Congo were analysed using the *COI* gene. Sequence analysis, based on 638 nucleotides, revealed a low polymorphism, with only two mutational sites defining three haplotypes namely H1, H2 and H3 ( Figure 2B). Consequently, this resulted in low haplotype diversity (Hd=0.24) and nucleotide diversity (π=0.00005) indexes ( Table 4). The most frequent haplotype, H1 (86.6%), was detected in all the locations. Supplementary Table 2 shows the haplotype distribution per location. The haplotypes H2 (10.2%) and H3 (3.2%) were found in three (Brazzaville, Ouesso and Oyo) and two (Brazzaville and Lefini) locations, respectively ( Table 4 and Figure 2). The dominant haplotype matches perfectly with the *COI* gene sequence deposited in GenBank that originated from China (KU738429.1). A higher genetic diversity was reported in Brazzaville where all the three haplotypes were reported. The haplotype network showed that each haplotype was separated from the others by one mutational step ( Figure 2). Overall, Tajima’s D (D=-0.294) and Fu’s Fs (Fs=-0.024) statistics were negative, but not statistically significant. Phylogenetic tree generated and analysed based on 445 nucleotides previously published in GenBank showed that the Republic of the Congo’s haplotypes were closely related to the sequences from China, Singapore, Papua New Guinea and Christmas Island ( Figure 3).

**Table 4.** Summary statistics for *COI* gene polymorphism in *Aedes albopictus* from the Republic of the Congo.

Locality	N	H	S	Hd	π (k)	D	D*	Fs	F*
Brazzaville	14	H1, H2, H3	2	0.692	0.0014 (0.890)	1.127 ^ns^	0.935 ^ns^	0.612 ^ns^	1.021 ^ns^
Lefini	15	H1, H3	2	0.133	0.0004 (0.266)	-1.490 ^ns^	-1.873 ^ns^	0.235 ^ns^	-1.844 ^ns^
Ngo	12	H1	0	0.000	0.0000 (0.000)	NC	NC	NC	NC
Gamboma	14	H1	0	0.000	0.0000 (0.000)	NC	NC	NC	NC
Oyo	12	H1, H2	1	0.303	0.0005 (0.303)	-0.195 ^ns^	0.752 ^ns^	0.297 ^ns^	0.533 ^ns^
Owando	11	H1	0	0.000	0.0000 (0.000)	NC	NC	NC	NC
Makoua	14	H1	0	0.000	0.0000 (0.000)	NC	NC	NC	NC
Ouesso	15	H1, H2	1	0.514	0.0008 (0.5143)	1.376 ^ns^	0.701 ^ns^	1.253 ^ns^	0.906 ^ns^
Pointe Noire	20	H1	0	0.000	0.0000 (0.000)	NC	NC	NC	NC
**Total**	**127**	**3**	**2**	0.246	**0.0005 (0.2952)**	**-0.294 ^ns^**	**0.662 ^ns^**	**-0.0238 ^ns^**	**0.417 ^ns^**

N, number of sequences; S, number of polymorphic sites; H, haplotype; Hd, haplotype diversity; π, nucleotide diversity; k, mean number of nucleotide differences; D, Tajima statistic; D* and F*, Fu and Li statistics; Fs, Fu statistic; NC, not computed; ns, not significant.

## Discussion

This study assessed the geographical distribution of *Ae. aegypti* and *Ae. albopictus* in the Republic of the Congo, revealing the co-occurrence of both species across the country. Analyses showed that the invasive species *Ae. albopictus* is the predominant species in all locations investigated except Brazzaville. The co-occurrence of *Ae. aegypti* and *Ae. albopictus* across the Republic of the Congo suggests that the environmental factors which prevail in the country are favourable for the development of both species. The presence of *Ae. albopictus* in the Republic of the Congo was confirmed in 2011 during the chikungunya outbreak in Brazzaville ^25^, suggesting its recent introduction. Indeed, previous studies in some central African countries such as Cameroon and Central Africa Republic showed that the co-occurrence of *Ae. aegypti* and *Ae. albopictus* is limited to the southern part of the country up to 6°N ^14, 15, 18^ suggesting that climate is a limiting factor for invasion. The predominance of the invading species, *Ae. albopictus,* over the indigenous species, *Ae. aegypti,* has been previously reported in areas where both species are found together in Central Africa. The ecological plasticity of *Ae. albopictus* has been suggested as the main cause of its adaptation to different environments ^9^, as well as its mating competitiveness in areas of sympatry with *Ae. aegypti* ^17, 38^. The prevalence of each species can vary according to the season in sympatric areas, as shown previously ^15, 39^, but was specifically linked to the duration of the dry season ^40^. Although, *Ae. aegypti* and *Ae. albopictus* have desiccant-resistant eggs, previous studies showed that *Ae. aegypti* eggs are more tolerant to high temperatures than those of *Ae. albopictus* ^41^. In both major cities where samples were analysed according to peri-urban and downtown environment, results revealed the predominance of *Ae. aegypti* in downtown areas but less in peri-urban areas. Similar findings to these were reported previously in Central Africa ^15, 18, 40^. These observations are consistent with former studies indicating the segregation of habitats in sympatric areas according to urban environmental gradients as the main factor responsible for the coexistence of *Ae. aegypti* and *Ae. albopictus* ^42, 43^. Used tyres were the most common container found positive for *Aedes* in all the locations. This is in accordance with previous studies in Central Africa showing that used tires are the main productive for both *Ae. aegypti* and *Ae. albopictus* ^13, 15^. However, the current study targeted mainly garages and tire shops to increase the chances of discovering immature *Aedes*.

The presence and predominance of *Ae. albopictus* across the Republic of the Congo can increase the risk of mosquito-borne arboviral diseases since *Ae. albopictus* has been found competent to transmit about 22 arboviruses ^44^. Notably, the emergence of dengue and chikungunya viruses in the human dominated environment in central Africa coincides with the invasion of *Ae. albopictus* in this area where it was found as the main vector ^13, 23, 25^. During previous studies in central Africa, *Ae. albopictus,* was found to be infected by Zika virus in natural conditions ^24^. It was also demonstrated that *Ae. albopictus* from Bangui in Central African Republic is able to transmit enzootic chikungunya virus strains ^45^.

A very low polymorphism of *Ae. albopictus* discovered in the Republic of the Congo in this study is in agreement with the previous studies using the *COI* gene in areas newly colonised by this species including some Central African countries ^15, 26, 46^. This low polymorphism is consistent with the recent introduction from a founder *Ae. albopictus* population or could be related to ubiquitous *Wolbachia* infection in populations of this species, as suggested previously ^47^. Brazzaville, the capital city of the Republic of the Congo would be probably the main entry point of *Ae. albopictus* in the country, as higher levels of polymorphism (all the three haplotypes recorded) were detected at this location. For instance, *Ae. albopictus* was reported for the first time in the Republic of the Congo in Brazzaville during a chikungunya outbreak which occurred in the country ^21, 25^. Phylogenetic analysis showed that the haplotype sequences from the Republic of the Congo are very close to the sequences isolated from populations originating from China, New Papua Guinea, Singapore and Christmas Islands. Primers used in this current study were not the same as those used in the previous study in Cameroon, Central African Republic and Sao Tome island. Therefore, the haplotypes in the current study cannot be compared with those detected in Central Africa. Nevertheless, these data indicate that the population of *Ae. albopictus* found in Central Africa probably originated from other tropical regions as previously suggested ^15, 26, 46^. It will be interesting to perform other studies at macro-geographic scale using other markers such as double-digest restriction-site-associated DNA sequencing to assess the genetic structure and the level of the gene flow between these populations.

## Conclusion

To our knowledge, this is the first study assessing the distribution of *Ae. aegypti* and *Ae. albopictus* in the Republic of the Congo since *Ae. albopictus* was reported in 2011. Both species were found across the country with *Ae. albopictus* predominating in almost all locations. Low genetic polymorphism of *Ae. albopictus* indicated a recent introduction into the country. The spread of the invading species across the country could change the epidemiology of arboviral diseases in the Republic of the Congo. Thus, it will be important to assess urgently, the vector competence of both *Aedes* species from the Republic of the Congo to prevent emergence or re-emergence of several arboviruses such as dengue, Zika and yellow fever viruses.

## Data availability

Sequence for *Aedes albopictus* haplotype H1 cytochrome oxidase subunit I ( *COI*) gene, partial cds; mitochondrial, GenBank accession number MH025948: http://identifiers.org/ncbigi/GI:1402399558.

Sequence for *A. albopictus* haplotype H2 cytochrome oxidase subunit I ( *COI*) gene, partial cds; mitochondrial, GenBank accession number MH025949: http://identifiers.org/ncbigi/GI:1402399560.

Sequence for *A. albopictus* haplotype H3 cytochrome oxidase subunit I ( *COI*) gene, partial cds; mitochondrial, GenBank accession number MH025950: http://identifiers.org/ncbigi/GI:1402399562.

## Supplementary material

**Supplementary Table 1. Outgroup sequences used for phylogenetic analysis.**

**Supplementary Table 2. H1, H2 and H3 haplotype distribution per location.**
