# Supplementary material for: Geographical distribution of Aedes aegypti and Aedes albopictus (Diptera: Culicidae) and genetic diversity of invading population of Ae. albopictus in the Republic of the Congo
Source: Wellcome Open Res. 2018 Dec 28;3:79. Originally published 2018 Jun 25. [Version 3] doi: 10.12688/wellcomeopenres.14659.3 (PMC6081977; doi:10.12688/wellcomeopenres.14659.3)
Supplement: Supplementary file 1 [file wellcomeopenres-3-16328-s0000.tgz › b79be691-ca54-4224-8586-918e133fa2e0.docx]

**Table S1. Outgroup sequences used for phylogenetic analysis**

| **Outgroup Name** | **Country** | **Accession Number** | **Authors** |
| --- | --- | --- | --- |
| China 6 | China | KC690910.1 | [[1](#_ENREF_1)] |
| Japan 1 | Japan | KC690919.1 | [[1](#_ENREF_1)] |
| Singapore 1 | Singapore | KC690922 | [[1](#_ENREF_1)] |
| Taiwan 1 | Taiwan | KC690913 | [[1](#_ENREF_1)] |
| Taiwan 2 | Taiwan | KC690915 | [[1](#_ENREF_1)] |
| Singapore 2 | Singapore | KC690924 | [[1](#_ENREF_1)] |
| USA 1 | USA | KC690896 | [[1](#_ENREF_1)] |
| USA 2 | USA | KC690943 | [[1](#_ENREF_1)] |
| USA 3 | USA | KC690934 | [[1](#_ENREF_1)] |
| Hawai 1 | Hawai | KC690957 | [[1](#_ENREF_1)] |
| Hawai 2 | Hawai | KC690958 | [[1](#_ENREF_1)] |
| Papua New Guinea 1 | Papua New Guinea | KY907296 | [[2](#_ENREF_2)] |
| Papua New Guinea 2 | Papua New Guinea | KC572351 | [[3](#_ENREF_3)] |
| Papua New Guinea 3 | Papua New Guinea | KC572356 | [[3](#_ENREF_3)] |
| Papua New Guinea 4 | Papua New Guinea | KY907438 | [[2](#_ENREF_2)] |
| China 2 | China | KC690898 | [[1](#_ENREF_1)] |
| China 3 | China | KC690908 | [[1](#_ENREF_1)] |
| Torres Strait Islands1 | Torres Strait Islands | KC572167 | [[3](#_ENREF_3)] |
| Torres Strait Islands2 | Torres Strait Islands | KC572191 | [[3](#_ENREF_3)] |
| Solomon Islands 1 | Solomon Islands | KY907378 | [[2](#_ENREF_2)] |
| Christmas Islands 2 | Christmas Islands | KY907297 | [[2](#_ENREF_2)] |
| Christmas Islands 1 | Christmas Islands | KY907391 | [[2](#_ENREF_2)] |
| Timor-Leste 1 | Timor-Leste 1 | KF042861 | [[3](#_ENREF_3)] |
| Timor-Leste 2 | Timor-Leste 2 | KF042876 | [[3](#_ENREF_3)] |
| Fiji | Fiji | KY907366 | [[2](#_ENREF_2)] |
| Papua New Guinea5 | Papua New Guinea | KC572319 | [[3](#_ENREF_3)] |
| Papua New Guinea6 | Papua New Guinea | KC572321 | [[3](#_ENREF_3)] |
| Papua New Guinea7 | Papua New Guinea | KC572267 | [[3](#_ENREF_3)] |
| Malaysia1 | Malaysia | KY907368 | [[2](#_ENREF_2)] |
| Malaysia2 | Malaysia | KY907373 | [[2](#_ENREF_2)] |
| Malaysia3 | Malaysia | KY907374 | [[2](#_ENREF_2)] |
| China 5 | China | KC690896 | [[1](#_ENREF_1)] |
| China 4 | China | KC690897 | [[1](#_ENREF_1)] |
| Singapore 1 | Singapore | KC690919 | [[1](#_ENREF_1)] |
| Taiwan | Taiwan | KC690912 | [[2](#_ENREF_2)] |
| China 8 | China | KU738429 | Unpublished |

References

1. Zhong D, Lo E, Hu R, Metzger ME, Cummings R, Bonizzoni M, et al. Genetic analysis of invasive *Aedes albopictus* populations in Los Angeles County, California and its potential public health impact. s*.* 2013; 8(7):e68586.

2. Maynard AJ, Ambrose L, Cooper RD, Chow WK, Davis JB, Muzari MO, et al. Tiger on the prowl: Invasion history and spatio-temporal genetic structure of the Asian tiger mosquito *Aedes albopictus* (Skuse 1894) in the Indo-Pacific. PLoS Neg Trop Dis*.* 2017;11(4):e0005546.

3. Beebe NW, Ambrose L, Hill LA, Davis JB, Hapgood G, Cooper RD, Russell RC, Ritchie SA, Reimer LJ, Lobo NF, et al. Tracing the tiger: population genetics provides valuable insights into the *Aedes (Stegomyia) albopictus* invasion of the Australasian Region. PLoS Neg Trop Dis. 2013;7(8):e2361.
