# Supplementary material for: Geographical distribution of Aedes aegypti and Aedes albopictus (Diptera: Culicidae) and genetic diversity of invading population of Ae. albopictus in the Republic of the Congo
Source: Wellcome Open Res. 2018 Dec 28;3:79. Originally published 2018 Jun 25. [Version 3] doi: 10.12688/wellcomeopenres.14659.3 (PMC6081977; doi:10.12688/wellcomeopenres.14659.3)
Supplement: Supplementary file 2 [file wellcomeopenres-3-16328-s0001.tgz › f4565497-f90c-4018-86ea-7c062ed1a6d9.docx]

Table S2. Distribution of COI gene haplotype detected across the Republic of the Congo

|  |  | | |  |
| --- | --- | --- | --- | --- |
|  | **Haplotype** | | |  |
| **Location** | H1 | H2 | H3 | All |
| Brazzaville | 6 | 5 | 3 | 14 |
| Ngo | 12 | 0 | 0 | 12 |
| Lefini | 14 | 0 | 1 | 15 |
| Owando | 11 | 0 | 0 | 11 |
| Oyo | 10 | 2 | 0 | 12 |
| Gamboma | 14 | 0 | 0 | 14 |
| Makoua | 14 | 0 | 0 | 14 |
| Ouesso | 9 | 6 | 0 | 15 |
| Pointe Noire | 20 | 0 | 0 | 20 |
| All | 110 | 13 | 4 | 127 |
