# Supplementary material for: Geographical distribution of Aedes aegypti and Aedes albopictus (Diptera: Culicidae) and genetic diversity of invading population of Ae. albopictus in the Republic of the Congo
Source: Wellcome Open Res. 2018 Dec 28;3:79. Originally published 2018 Jun 25. [Version 3] doi: 10.12688/wellcomeopenres.14659.3 (PMC6081977; doi:10.12688/wellcomeopenres.14659.3)
Supplement: Peer review history

# Peer review: Referee response for version 2

**DOI:** 10.21956/wellcomeopenres.16070.r33461

**Published:** 2018-08-31

**Authors:** Julien B. Z. Zahouli

**Affiliations:** 1 Swiss Tropical and Public Health Institute (Swiss TPH), Basel, Switzerland

**1) General comments**

 Kamgang *et al*. conducted this study with the overall aim of assessing the geographical distribution of *Aedes aegypti* and *Aedes albopictus* and the genetic diversity of *Ae. albopictus* in the Republic of Congo. Specifically, the authors sampled immature *Aedes *mosquitos among their usual breeding sites in 9 sites across several eco-regions, reared immature to adult stage for species morphological identification, and analyze the diversity of *Ae. albopictus* using CO1 gene method.

 This study merits a careful and particular attention. While it is not novel to describe the Aedes mosquito species in Africa, this study is the one focusing on the invading Asian tiger *Ae. albopictus* which has been newly introduced into the African continent by the means of transportation and trade.  The authors identified three Aedes species ( *Aedes aegypti*, *Aedes albopictus* and *Aedes simpsoni*) that may have an important impact on public health as they may carry and transmit pathogens. The study shows some clear results, which include the variation of among mosquito species abundance of *Aedes aegypti* and *Aedes albopictus* and genetic diversity of *Ae. albopictus* species as a function of location. The outcomes were represented as texts, figures and tables. The outcomes of this work could be explored for better understanding of the distribution of *Aedes aegypti* and *Aedes albopictus* species that can influence the transmission of arboviruses to humans and may contribute the control arboviral diseases in the Republic of Congo.

 Despite its strength, there are a number of weaknesses and gaps to the study that need to be addressed. The study is inherently limited to represent the species and specimens of non- *Aedes* mosquitoes and some features of the containers. The authors did not characterize the larval habits by looking at some ecological features of the containers. Some important characteristics such as the exposure of containers to sunlight, the presence or absence of microbial food inputs, air temperature, rainfall, temperature of water, turbidity of water, color of containers and some biological interactions (predation, competition, sympatry…) involving predators, competitors and other associations among mosquito  larvae were not reported. The report of these factors may probably help to better understand the difference in ecological distribution and patterns between *Aedes aegypti* and *Aedes albopictus* species. The authors talked about the displacement of *Aedes aegypti* due to the presence of *Aedes albopictus* in the abstract without clearly indicating the strength of the displacement and presenting data showing this important ecological phenomenon.  The objective of this study is not clear to me. The hypothesis of this study was not provided. The authors should provide the objective and hypothesis of their works.

 The writing throughout the manuscript is good to me. However, the writing needs to be improved in some places. In addition, as the authors did not sample the ecological characteristics of sampling areas and containers, they discussed their findings on the basis of the findings of other studies – this seems to consider them as speculations – the authors should focus on the own data, discuss them on the basis of their own data sampled and drawn inferences after comparing the data with previous studies.

 Ultimately, above all, more importantly are the paper's weaknesses that should be addressed. I have provided more detailed and specific comments below.

**2) Specific comments**

****

**Background**

 Line 3 (It….associated): Please replace “was” with “has been”

 Line 4-5 (how ….. diseases): It seems that this paper did not focus on the epidemiology of arboviral diseases, but the ecology of Aedes species. Please, it suggested that the authors delete or provide clearer explanation.

**Abstract**

**Background**

 Line 2 (in 2017): It suggested that the authors clarify the period (month) of the study. The authors should write the date at the start of the sentence or at the end of the sentence, but not at the middle. Please, correct.

**Results:**

 The authors stated that *Ae. albopictus* has been newly introduction to Congo and that *Ae. albopictus* has displaced *Ae. aegypti*. I am concerned – please, what was the distribution of Ae aegypti before the introduction of *Ae. albopictus*? Please, how do the authors know that *Ae. Albopictus* is displacing Ae. aegypti since I did not see data on such an ecological issue in this manuscript. Please, correct.

 Line 4-5 (suggesting …….across Congo): The portion of the sentence seems to be a discussion – may I suggest that the authors displace it into the Conclusion.

 Line 9-10 (supporting …….the Congo): The portion of the sentence seems to be a discussion – may I suggest that the authors displace it into the Conclusion.

 Line 11-12 (likely …….trade links): The portion of the sentence seems to be a discussion – may I suggest that the authors displace it into the Conclusion.

**Introduction**

 Please replace “zika” with “Zika” in the whole document (please use a capital letter “Z”

 Line (This rapid …….caused mainly…): Please, replace “was” with “has been”

 Line (This suggests …… in the region): This sentence is not clear to me – may the authors clarify?

 May I suggest that the authors clearly provide  the objective and hypothesis of this study?

**Methods**

 Line (The humid tropical climate…..in the southwest): It seems that words are missing here – maybe “is found”?

 Line (In Brazzaville…..downtown and suburban…). How did the authors make the difference between “downtown” and “suburban”? What were the criteria?

 Line (with at least one Aedes larvae or pupae) – please, may the authors replace “larvae” with “larva” (singular form!) and “pupae” and “pupa” (singular form)?

 Line (….stage for morphological identification.): please, may the authors specify the determination key used for morphological identification and the reference?

 Lines (The comparisons between …………chi-square test): Is this a statistical analysis of the data? I am not sure that this corresponds to the title of this paragraph – usually, the statistical analysis of data is separated from the lab works – may the authors write an independent paragraph clearly describing the statistical analysis procedures? It will be interesting if the authors perform geospatial analysis of the data to clearly show the distribution of *Ae. albopictus* and *Ae. aegypti* across sampling areas.

 Lines (in each selected location ALL containers, ….were recorded) – I am not sure that it is possible to sample ALL containers in a given site – usually, only readily visible and accessible containers can be sampled – please, it is suggested that the authors reword this sentence?

 It appears that the authors did not sample the characteristics of containers and habitats, but only the mosquito immatures – If so, how will they explain the distribution of species? It should be important to collect information on the biotic and abiotic characteristics/variables of the sampling sites and breeding containers.

 May the authors explain or justify the importance of using *CO1* method to assess the origin of *Ae. albopictus* instead of other technique? What are the advantages of using *CO1* method?

**Results**

 As I said previously, while the authors stated that ALL containers were sampled in such large areas, only 640 containers were sampled? In my experience, it is not possible to sample all containers in such large areas.

 Were the breeding sites and land cover not characterized? The characteristics of the breeding containers and land cover are likely to affect species distributions. It is suggested that the authors described the characteristics of *Aedes* breeding sites and land covers surrounding the containers.

 It is suggested that the authors add a paragraph focusing on the distribution of both main Aedes species, *Ae. aegypti* and *Ae. albopictus*, among the containers samples? May the authors also specify the proportions of containers that both species co-habited?

 Table 3: The overall proportions of *Ae. aegypti* (27.72%) and *Ae. albopictus* (72.28%) in the Table 3 does not match with the proportions indicated in the text (In total, 6,684 specimens …….. comprising 72.24%.......27.70%....). Please, explain or correct these differences.

 I am concerned: non- *Aedes* mosquitoes were not collected? Usually, *Culex* species, sometimes *Anopheles* species and other predatory larvae of mosquitoes inhabit *Aedes* breeding sites… These species are likely to alter the ecology and distribution of *Ae. aegypti* and *Ae. albopictus*. Please, it is suggested that the authors add other mosquito species if they collected.

**Discussion**

 Line (This study has assessed….): please, it is suggested deleting “has”.

 Line (Analyses showed…..is….Brazzaville): please, may the authors replace “is” with “was” (past simple / preterit) – as the authors are reporting their own data?

 Line (The co-occurrence of *Ae. aegypti* …….environmental factors……..both species) – please, may the authors explain which environmental factors that influence the co-occurrence of both Aedes species? I did not see a clear link/association/correlation between such environmental factors and *Ae. aegypti* and *Ae. albopictus* distribution. It should be good if the authors sampled and reported the environmental variables (vegetation, land cover, temperature, humidity, rainfall, season, sunlight radiation, shade, nature of water of the containers, type of breeding sites, foods, predators, competitors,)  in each specific location and look at the relationships between these variable with the presence/or abundance of the species. May the authors provide such relationship between environmental parameters and the occurrence of *Aedes* species?

 Line (The ecological plasticity….): The data did not clearly report the ecological plasticity/elasticity in *Ae. albopictus* which means that the species colonize different habitats (for instance, vegetated/open environment), containers (natural, peridomestic, domestic…), land covers (rural, urban, forests, savannah…)…. Please, may I suggest that the authors report such findings in the Results season to support their discussion?

 Line (The prevalence……….peri-urban areas) - Here the authors seem to link the difference in the distribution of Aedes species between downtown and peri-urban environment to eggs tolerance/desiccation to temperature variations. But, as they did not record the temperature among both environments, it is difficult to understand such an inference… Why not other environmental parameters such as human density, human activities/behaviors, shade/sunlight, the nature of breeding sites or containers?

 Line (This is in accordance with ……….the main productive for both Ae. aegypti and Ae. albopictus): It seems that some words are missing in the sentence – it appears to be “…main productive -breeding sites- for..”

 Line (However, the …………..garages and tire shops…) – may I suggest that the authors in the Methods that the larval collection was performed mainly in the garages and tire shops? It seems that the authors were mixing UK/British spelling and US spelling! As the overall manuscript is written using UK English, they should write “tyre” /UK English) instead of “tire” (US English). Please, correct in the whole document.

 Line (…. *Ae. albopictus* **,** was found to be infected….) – please, it suggested deleting coma “,”.

**Conclusion**

 Line (…SEVERAL emergence or re-emergence of arboviruses….) – may I suggest “….. emergence or re-emergence of SEVERAL arboviruses…”

 Line (Assessing the susceptibility……….future outbreaks) – yes, but I do see the link between the current study and insecticide susceptibility issue. Please, it is suggested to delete this sentence.

I have read this submission. I believe that I have an appropriate level of expertise to confirm that it is of an acceptable scientific standard.

# Response

**Published:** 2018-12-19

**Authors:** Basile Kamgang

**Affiliations:** Centre for Research in Infectious Disease, Cameroon

Our study aim is to assess the geographical distribution of *Ae. aegypti* and *Ae. albopictus* in the Republic of Congo as well as the genetic diversity of invading species. In this study, sampling was focused mainly in garages and tire shops due to the previous data reported in Central Africa demonstrating that Ae. *aegypti* and *Ae. albopictus* breed mainly in the used tires. Thus, this strategy was used to increase the chance collected immature stage of Aedes mosquitoes in domesticated environment.

 In each city, we sampled in downtown and in suburb basing also to the previous result in Central Africa showing that in the cities in which both species are found, *Ae. aegypti* is most prevalent in down town with higher building density whereas Ae. albopictus is rather more abundant in periphery area of the city surrounded by the vegetation (kamgang et al, 2010; Kamgang et al., 2013; Kamgang et al., 2017).

 I wanted to precise that the aim was not to characterize the breeding sites of Aedes, this can explain why the data on presence of vegetation, sun light exposure, color of breeding sites, distance between plants and containers,… were not recorded.

 The proportion of Aedes in the text included four *Ae. simpsoni* collected in Brazzaville.

 Mitochondrial DNA gene (COI) used here has been extensively used to assess genetic diversity of *Ae. albopictus* even it seem not really suitable in the region recently invaded due to their low polymorphism.

 It true that there is no previous data indicated the abundance of *Ae. aegypti* in the Republic of Congo meanwhile taking other the studies implemented in Central African Republic and Cameroon showing that *Ae. albopictus* became dominant species some years after his introduction, we can conclude that the same situation is ongoing in the Republic of Congo. In general, all the hypothesis did in the discussion section are based on the findings in the previous works in the region.

# Peer review: Referee response for version 1

**DOI:** 10.21956/wellcomeopenres.15961.r33442

**Published:** 2018-08-07

**Authors:** Sébastien Boyer

**Affiliations:** 1 Unit of Medical Entomology, Institut Pasteur of Cambodia, Phnom Penh, Cambodia

The study is important and essential in the Public Health domain, especially in the Republic of Congo where data on vectors are missing, and where doing field study is not that easy.

 Even knowing it is difficult to do field studies, from a scientific point of view, there is no replicate in this experiment. The authors went into the field one time in May 2017 and one time in November 2017. It should not be count as two independent samples of the same sites, but it should be considered as one sample during one season, and one sample during the other season. It is better to have a representation of the geographic distribution in the both season to observe if there is a variation than to have some chi-square tests that are not really useful here. It didn’t deserve the study. As I mentioned earlier, I truly think that the data are important here, including the percentage of positive container for each species, but is should be presented per season also, with no replicate.

 For representing the data illustrated in the Figure 2, it should be better to represent it by chart by city. Here we didn’t see very well what is the percentage of H1, H2 and H3 in Brazzaville for example. It is the same problem for the proportion of H1 and H2 in Ouesso, H1 and H2 in Oyo and H1and H3 in Lefini. By the way, it is very interesting to observe that these 3 haplotypes are nearby Asia and SouthEast Asia haplotypes.

 In total, the study is well realized, and the conclusions are directly dependent of the results. But I suggest 2 only minor changes regarding the presentation of the results, especially for the sampling in May and November.

 I also suggest to replace the Figure 2 by a map with the % of each haplotype (circle or histograms) on each city.

I have read this submission. I believe that I have an appropriate level of expertise to confirm that it is of an acceptable scientific standard.

# Response

**Published:** 2018-08-07

**Authors:** Basile Kamgang

**Affiliations:** Centre for Research in Infectious Disease, Cameroon

Thank you so much for your comments. Mosquitoes were sampled in May in 8 locations (Brazzaville, Lefini, Ngo, Owando, Makoua, Ouesso, Oyo and Gamboma, and in November in Pointe Noire only) corresponding to rany season in each location. Precision was make in the manuscript.

 The number of haplotypes detected per city is presented in supplement Table S2.

# Peer review: Referee response for version 1

**DOI:** 10.21956/wellcomeopenres.15961.r33460

**Published:** 2018-07-23

**Authors:** Yao Lucien Konan

**Affiliations:** 1 Vector Control Department, National Institute of Public Hygiene , Abidjan, Cote d'Ivoire

The work is clearly written in a precise way with recent references used.

 At the method level, it should be noted the months of mosquito collection were indicated without the seasonal correspondence in the different localities.

 In the results, the authors mention productivity of used tires while the larvae and pupae found in the different containers inspected were not counted. They must replace "productive" with "positive".

I have read this submission. I believe that I have an appropriate level of expertise to confirm that it is of an acceptable scientific standard.

# Response

**Published:** 2018-07-23

**Authors:** Basile Kamgang

**Affiliations:** Centre for Research in Infectious Disease, Cameroon

I agree with the reviewer that the season was not indicated.

Mosquitoes were collected in May and November 2017 corresponding to the rainy season in nine locations in the Republic of the Congo across the north-south transect.

It is true that the number of pupae and larvae has not been counted, but the number of mosquitoes emerging from each type of larval habitat has been counted.
